# Research trends of music in children with autism: a bibliometric analysis

**DOI:** 10.3389/fpsyt.2025.1553883

**Published:** 2025-08-12

**Authors:** Ye Tao, Chun Yu

**Affiliations:** ^1^ Department of Special Education, College of Child Development and Education, Zhejiang Normal University, Hangzhou, China; ^2^ Department of Public Education, College of Child Development and Education, Zhejiang Normal University, Hangzhou, China

**Keywords:** autism spectrum disorder, music, bibliometrics, children, Web of Science Core Collection

## Abstract

**Introduction:**

The impact of music on children with autism—a condition marked by deficits in social interaction, communication, and behavior—has become a significant area of research. This study investigates current trends, key contributors, and emerging interests regarding music’s effects on this population.

**Methods:**

A comprehensive review of publications from 1953 to 2024 on the influence of music on children with autism was conducted using the Web of Science Core Collection. Bibliometric and visual analyses were performed with VOSviewer, CiteSpace, and R version 4.3.3.

**Results:**

A total of 411 research papers were identified, with significant publication growth noted post-2009. The leading countries in this research include the United States, the United Kingdom, Canada, China, and Australia. McGill University ranked as the most prolific institution (23 publications), followed by the University of Montreal (17) and Vanderbilt University (12). The Journal of Autism and Developmental Disorders is the most influential journal, with an h-index of 19 and 1,706 citations. Professor Christian Gold emerged as the top author, with 12 papers totaling 599 citations. Key keywords included “children,” “autism,” and “therapy,” with a noted increase in terms like “social skills,” “communication,” and “engagement” since 2020.

**Conclusion:**

This study highlights music’s potential to enhance social and communication skills in children with autism. Future research should explore the long-term effects of music therapy on language, cognition, and behavioral outcomes, as well as its role in improving engagement in educational and therapeutic settings.

## Introduction

Autism is a neurodevelopmental disorder that primarily affects social interaction, communication abilities, and behavioral patterns (1, 2). Individuals with autism may face challenges in social interaction, verbal communication, and understanding non-verbal cues (3, 4). They often exhibit repetitive behaviors or an intense focus on specific interests. Additionally, individuals with autism may have atypical sensitivities to sensory stimuli (5, 6). With appropriate treatment and support, including behavioral therapies, educational support, speech and language therapy, occupational therapy, and social skills training, many individuals with autism can make significant progress in social and life skills (7).

Music, in particular, has emerged as a promising tool for improving the quality of life for children with autism. Some studies show that music interventions can significantly enhance social interaction, helping children with autism better communicate with others, increase eye contact, and improve social responsiveness (8, 9). It can also improve their communication skills, especially in non-verbal communication and symbolic expression (10). Furthermore, music can help regulate behavioral issues, reducing repetitive behaviors and negative emotional reactions by using rhythm and melody to assist in behavioral control (11, 12). Lastly, music provides a safe outlet for emotional expression and regulation, promoting emotional well-being (12, 13). An increasing number of researchers are engaging in studies on the impact of music on children with autism.

Bibliometrics is a branch of scient metrics that uses quantitative analysis of books, articles, and other forms of publications to study the patterns of scientific communication and academic research (14). It typically involves statistical analysis of citations, publication trends, research collaboration networks, and journal impact to uncover the development status, research hotspots, and frontiers in a specific field (15). Xiang Feng and colleagues conducted a bibliometric study that comprehensively analyzed research on autism signaling pathways between 2013 and 2023, highlighting the importance of molecular mechanisms, genetic studies, gut microbiota, the impact of stress, maternal immune activation, and neurodevelopment in autism research and interventions (16). There are also studies that provide a comprehensive bibliometric overview of the relationship between autism spectrum disorder in children and gut microbiota (17). However, in the field of research on the impact of music on children with autism, no comprehensive bibliometric analysis has been conducted yet. Therefore, this study aims to identify research hotspots and trends in the impact of music on children with autism using bibliometric analysis.

## Materials and methods

### Literature search and selection

A literature search was conducted in the Web of Science Core Collection (WoSCC), which offers a comprehensive and interdisciplinary database to identify literature on music interventions for children with autism spanning from 1953 to 2024. The search was executed on June 12, 2024, ensuring that the most recent data was included. The search query was as follows: ((((TS=(Autism)) OR TS=(Autistic)) OR TS= (Kanner Syndrome)) AND ((TS=(Music)) OR TS=(Musical))) AND (((TS=(Child)) OR TS=(Children)) OR TS=(Kid)). The search was limited to English-language articles.

### Statistical analysis and visualization

Microsoft Excel was employed to organize and analyze the retrieved literature data, identifying and calculating bibliometric indicators including annual publication counts, citation frequencies, average citation rates, as well as details of journals, impact factors, publication countries/regions, institutions and authors. For visualization, two bibliometric software tools, VOSviewer (version 1.6.20) and CiteSpace (version 6.3.R1), along with R 4.3.3, were employed, each serving specific analytical purposes. VOSviewer mapped institutional collaboration, author collaboration, co-authorship, citations, and co-citations (18). Keyword co-occurrence analysis was also conducted using VOSviewer to visualize complex academic relationships and identify key research themes. CiteSpace was applied to detect bursts of keyword usage, indicating emerging trends and research frontiers. Keyword co-occurrence analysis in CiteSpace used the following parameters: time slicing from January 1994 to May 2024, node types set to keywords, and a threshold (top N in each slice) of 5. Pruning was achieved using the pathfinder and merged network methods. In the visual representations, node sizes represented the number of publications, line thickness reflected the strength of the connections, and different node colors indicated various clusters or time periods. To quantify academic impact, metrics such as the H-index, G-index, and M-index were employed, as referenced in existing literature (19, 20). These metrics are critical for assessing the contributions of researchers and predicting future scientific output. Additionally, Journal Citation Reports (JCR) quartiles and Impact Factors (IF) were utilized to evaluate the significance and influence of journals publishing the relevant studies.

## Results

### Results of literature screening

**Figure 1:**
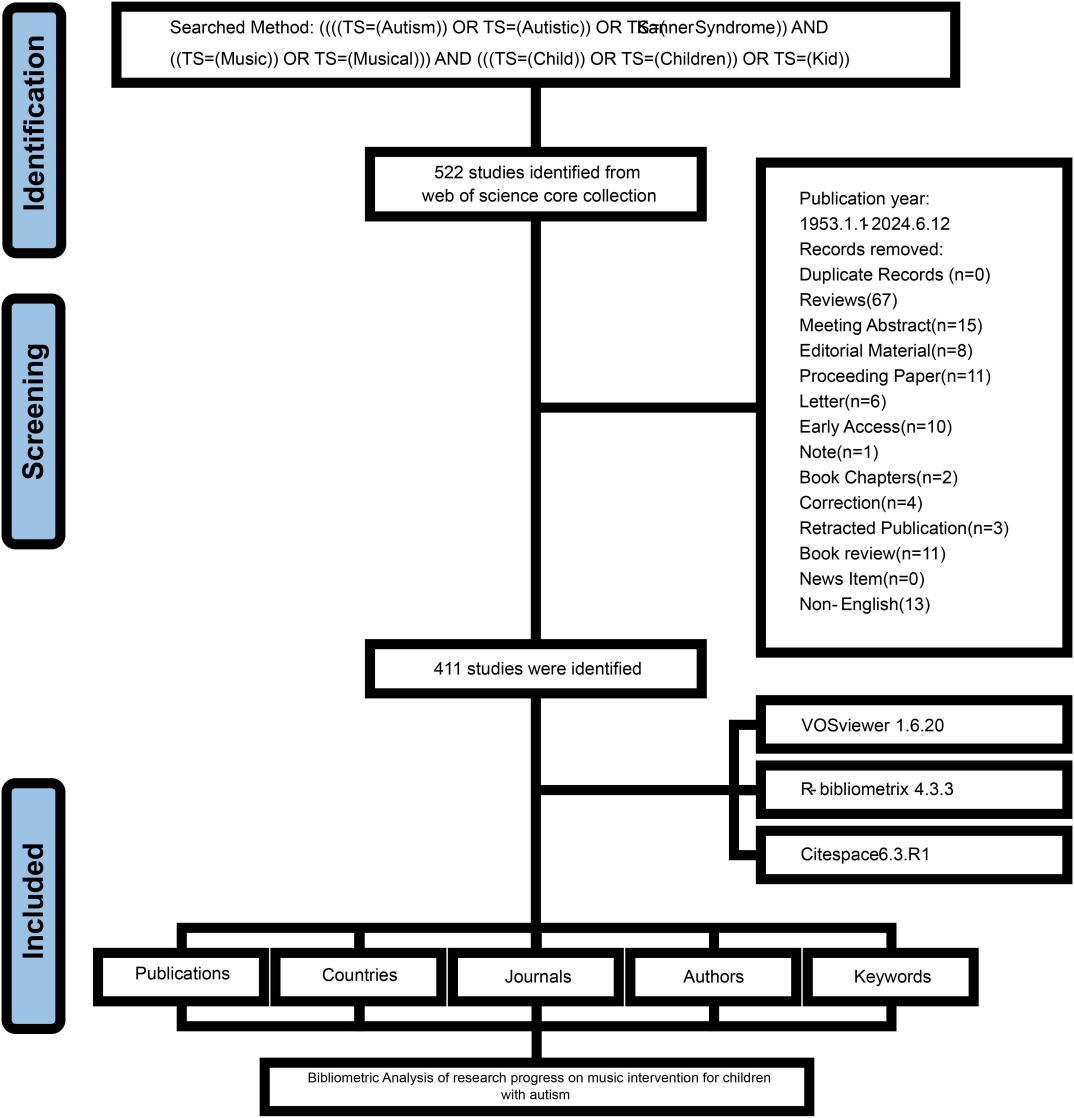
Flowchart of the literature screening process.

As illustrated in **Figure 1**, there have been a total of 522 publications on the impact of music on children with autism from 1953 to 2024. Among these, research articles constitute the most significant portion, comprising 411 publications, or 78.7% of the total. The remaining publications include several key types, such as reviews, conference abstracts and papers, editorial materials, and book chapters, which together account for about 20% of the overall output.

### Overview of publications

**Figure 2:**
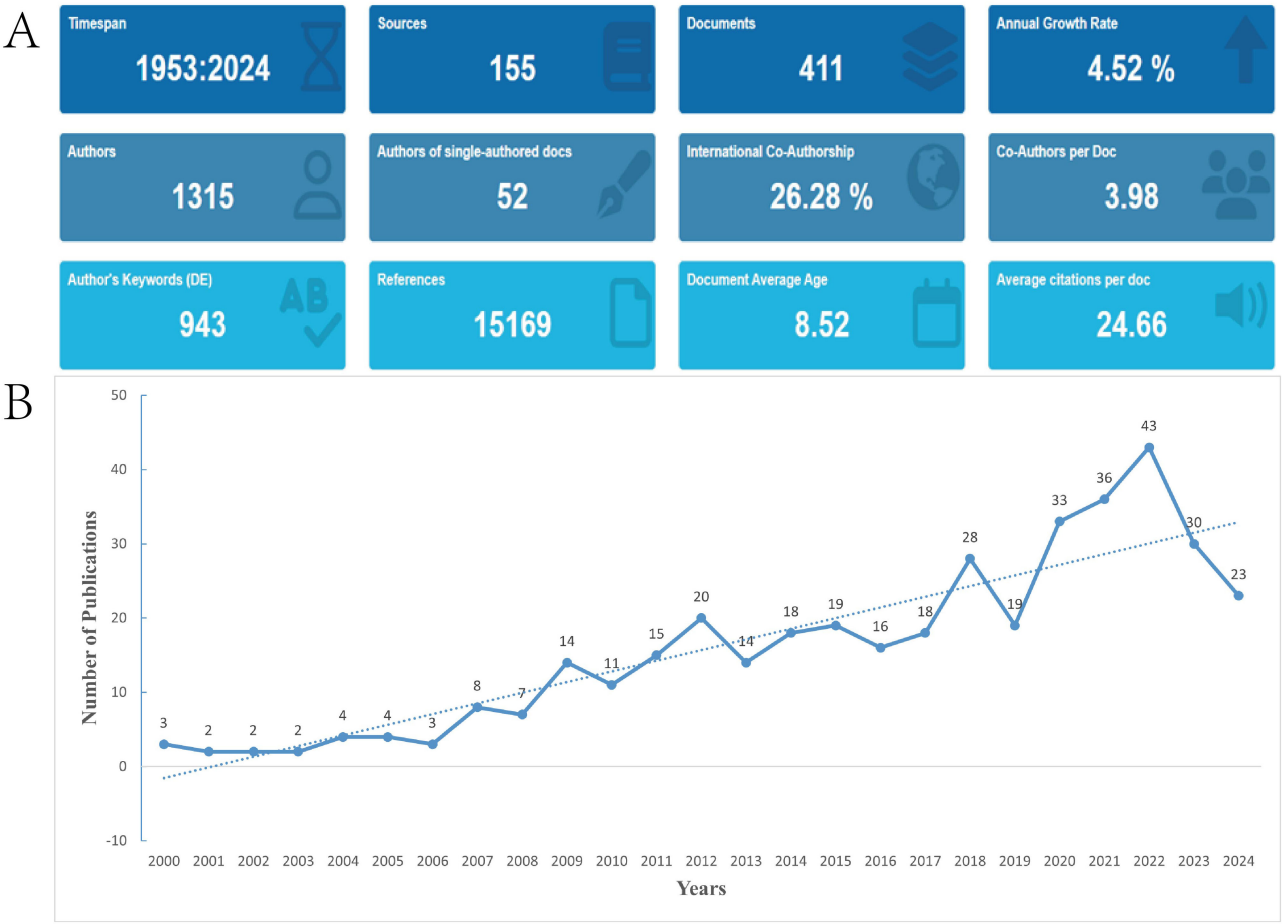
Analysis of general information. **(A)** Summary Information of the included studies. **(B)** Growth trend of the publications on the impact of music on children with autism.

This study examined 411 publications, all of which were articles. Our analysis revealed that 1,315 authors from 983 institutions across 202 countries/regions contributed to these 411 manuscripts. These works were published in 155 journals and cited 15,169 references (**Figure 2A**). **Figure 2B** depicts the trend in the number of publications per year from 2000 to 2024. Starting in 2007, the number of publications gradually increased, with a notable rise after 2009. Peaks were observed in 2012 and 2018, with 20 and 28 publications, respectively. A significant increase occurred in 2021, with 36 publications, reaching the highest point in 2022 with 43 publications.

### Analysis of countries

**Figure 3:**
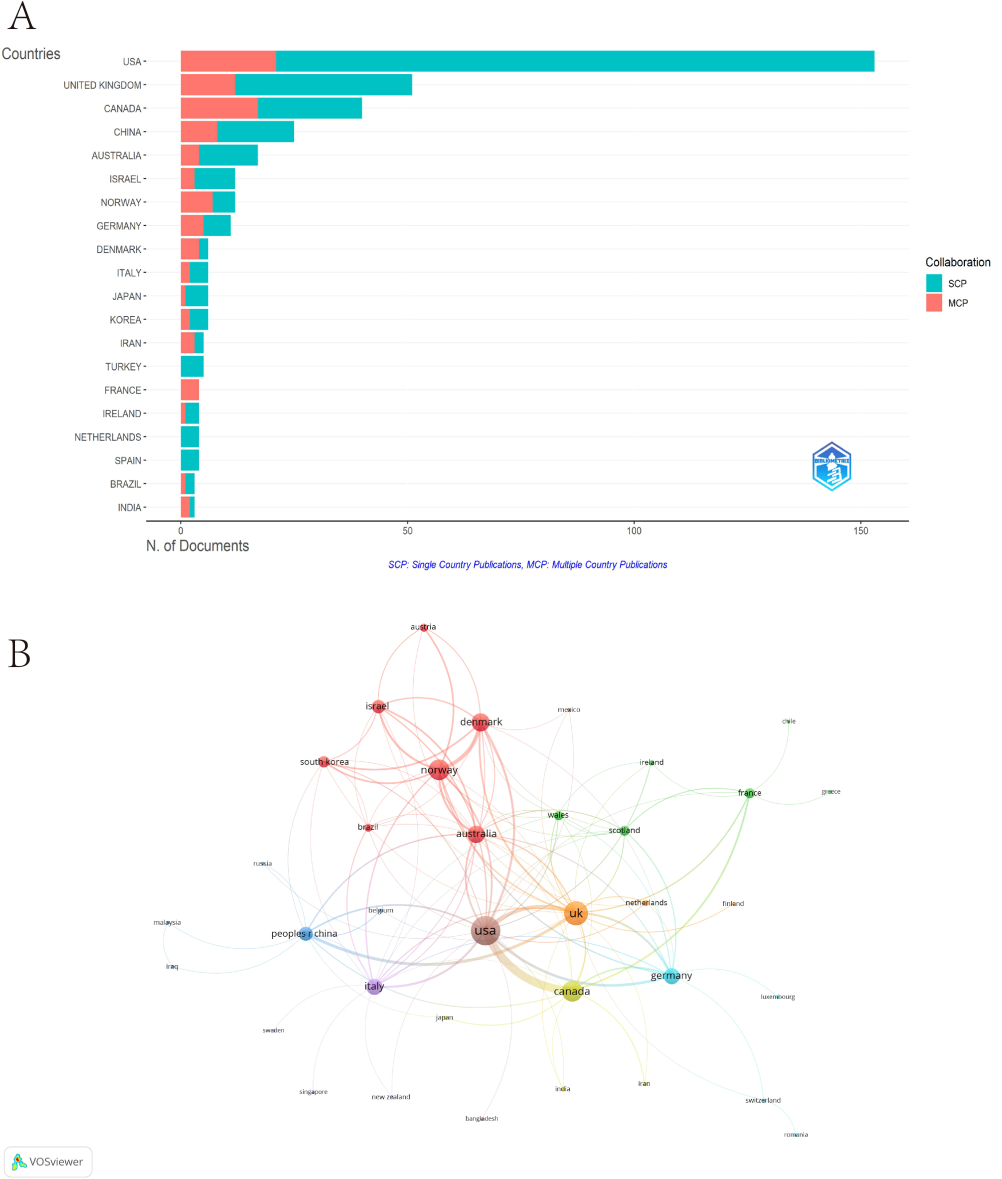
Analysis of countries. **(A)** The top 20 countries. **(B)** Visualization networks of countries.

The top contributing countries are the United States (153 articles), the United Kingdom (51), Canada (40), China (25), and Australia (17). Korea, Denmark, and Canada have the highest average citation rates, with scores of 56.8, 47.5, and 39.5, respectively. Most articles were single-country publications, with notable international collaboration ratios. The Multiple Country Publications (MCP) Ratio was highest for France (100.0), followed by Denmark (66.67) and Iran (60.0) (**Supplementary Table 1**) (**Supplementary Table 1**, **Figure 3A**). Analysis revealed that 46 countries engaged in collaborative research, each with at least one publication. The collaboration network highlights that the United States leads in collaboration, followed by the United Kingdom and Norway, with total link strengths of 77, 57, and 44, respectively (**Figure 3B**).

### Analysis of journals

**Figure 4:**
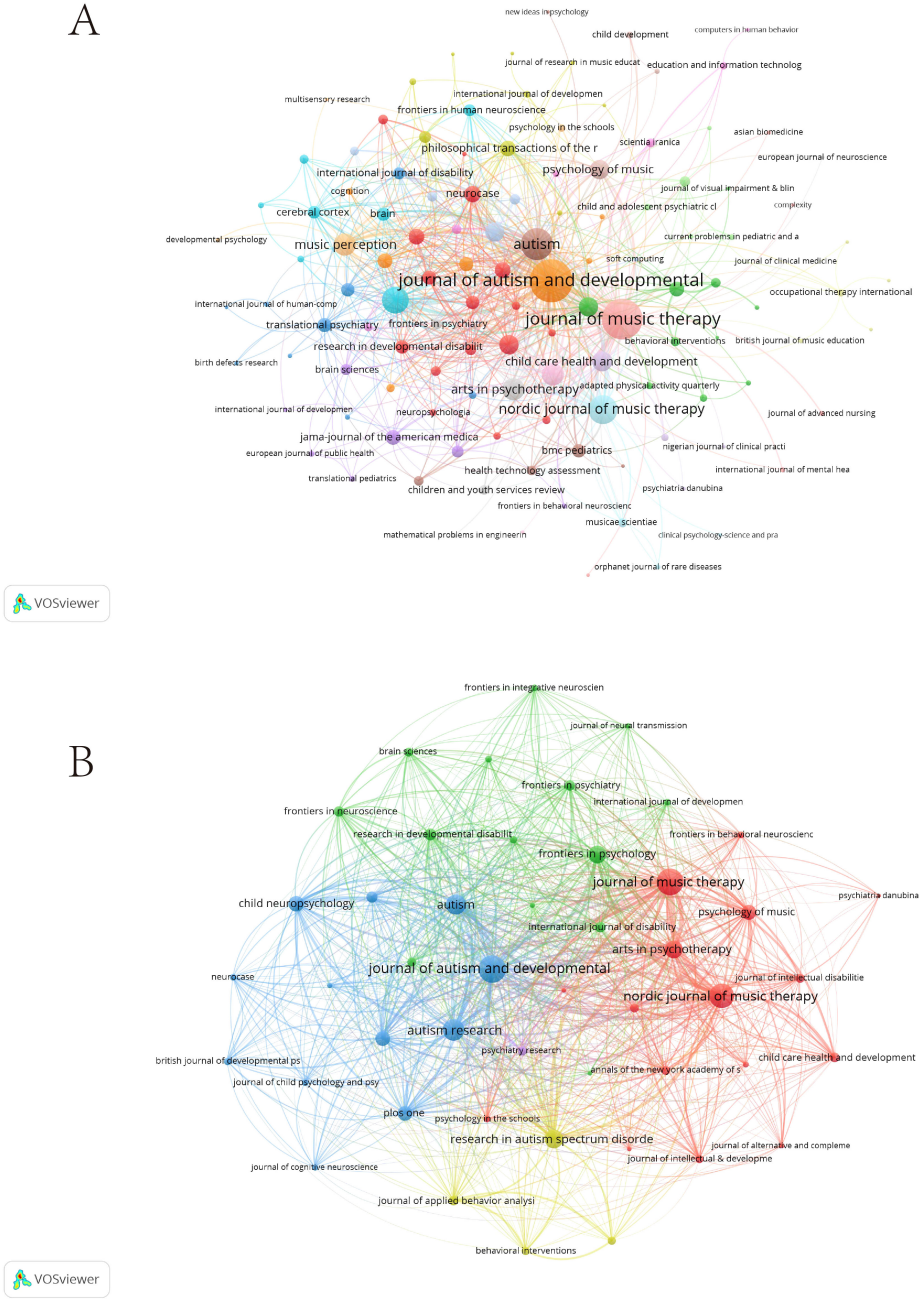
Analysis of journals. **(A)** Co-citation network of journals. **(B)** Coupling network of journals.

The 411 articles were published in 155 journals. The top 20 journals accounted for 241 articles, representing 58.64% of the total publications (**Supplementary Table 2**). The highest H-index journal was *Journal of Autism and Developmental Disorders* (H-index=19, TP=36, TC=1706), followed by *Journal of Music Therapy* (H-index=19, TP=41, TC=661) and *Autism* (H-index=11, TP=14, TC=421). The co-occurrence networks of journals contain 139 with at least 1 occurrence. Three key journals have the highest total link strength: *Journal of Autism and Developmental Disorders* (409), *Journal of Music Therapy* (367), and *Autism* (217) (**Figure 4A**). The coupling networks of journals contain 47 with at least 2 couples. The three journals with the highest total link strength are *Journal of Autism and Developmental Disorders* (5,598), *Journal of Music Therapy* (5,101), and *Nordic Journal of Music Therapy* (4,191) (**Figure 4B**).

### Analysis of authors

**Figure 5:**
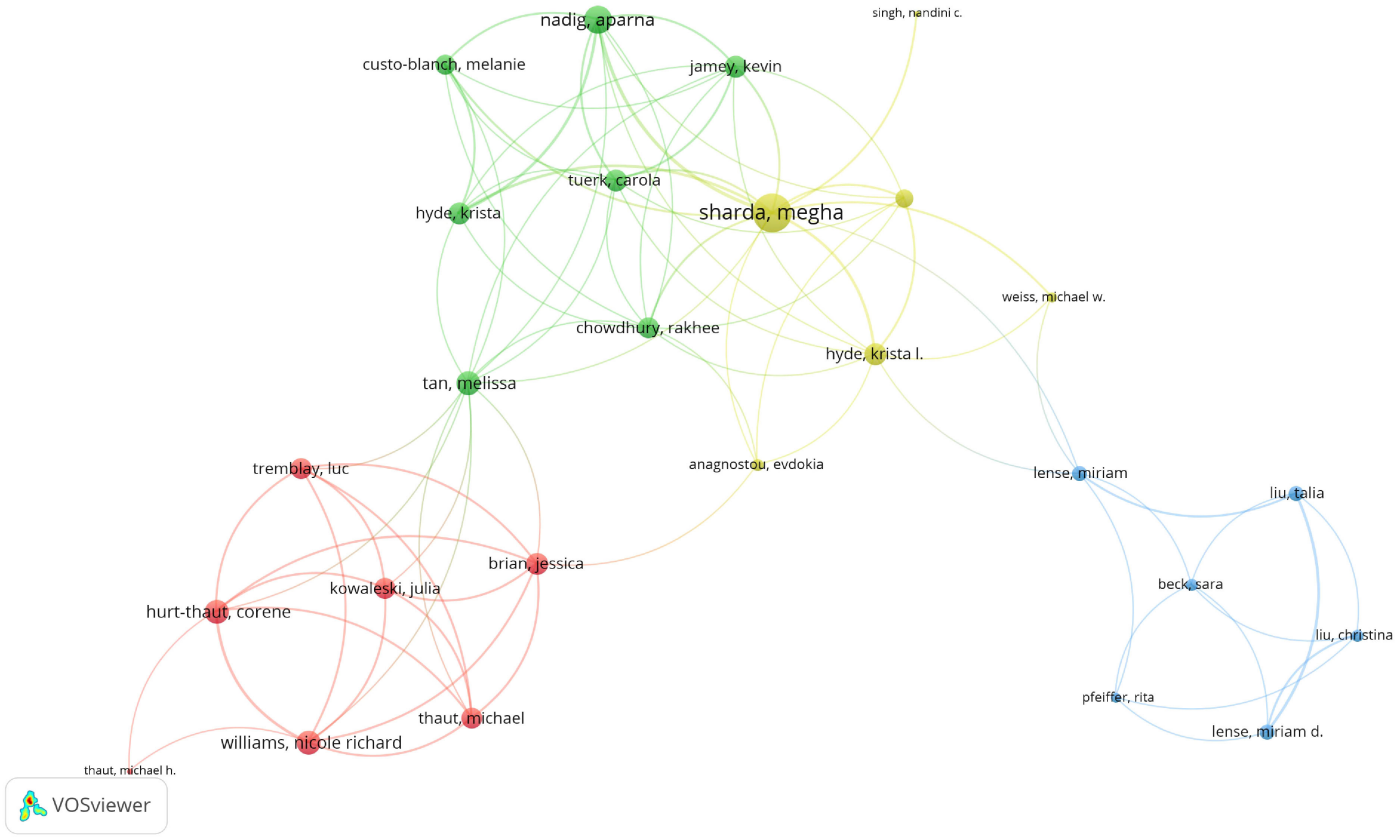
Visualization networks of author collaborations.

**Supplementary Table 3** presents the academic contributions of the top 20 high-impact authors in the field of music’s impact on children with autism. Collectively, they have published 110 articles with a total of 1,403 citations. Gold, Christian was the most cited author (cited 599 times, h-index=9, TP=12), followed by Heaton, Pamela (cited 509 times, h-index=10, TP=14) and Sharda, Megha (cited 218 times, h-index=6, TP=9). In addition, collaborations with a minimum of 2 articles show that Gold, Christian has the highest number of collaborations with other authors (42), followed by Geretsegger, Monika (28) and Sharda, Megha (27) (**Figure 5**).

### Analysis of institutions

**Figure 6:**
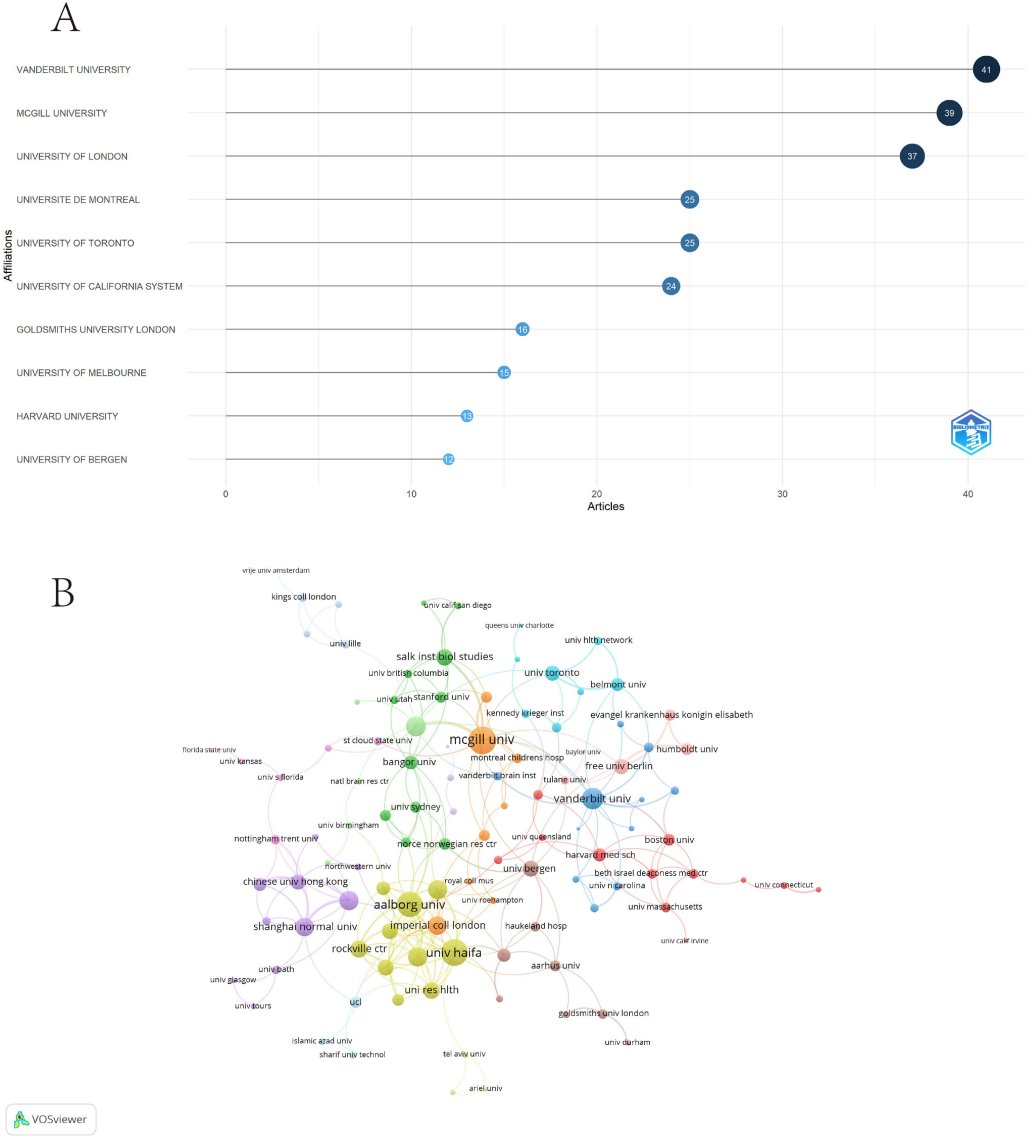
Analysis of institutions. **(A)** The top 10 institutions by article count and rank. **(B)** Visualization networks of institution collaborations.

Vanderbilt University leads with 41 articles, followed closely by McGill University with 39 articles and the University of London with 37 articles. Other notable institutions include Université de Montréal and the University of Toronto, each with 25 articles, and the University of California System with 24 articles (**Figure 6A**). The collaboration network in the **Figure 6B** shows several key clusters. Vanderbilt University and McGill University are central nodes in the network, indicating strong collaborative ties. The orange cluster consists of prominent institutions such as McGill University, Vanderbilt University, and the University of Toronto, primarily involving North American collaborations. The green cluster features leading research institutions such as Stanford University, the University of Sydney, and the Salk Institute for Biological Studies, showing a strong mix of international collaborations. The yellow cluster, centered around Aalborg University and the University of Haifa, reflects collaborations between European and Middle Eastern institutions. Meanwhile, the red cluster, led by institutions such as Humboldt University and Free University of Berlin, highlights significant collaborations within Europe. Other regional networks, such as the purple cluster, connect universities in Asia, including Chinese University Hong Kong and Shanghai Normal University, showing strong regional collaborations. In terms of total link strength, McGill University (27), University of Haifa (26), and Aalborg University (24) are the most central.

### Analysis of keywords

**Figure 7:**
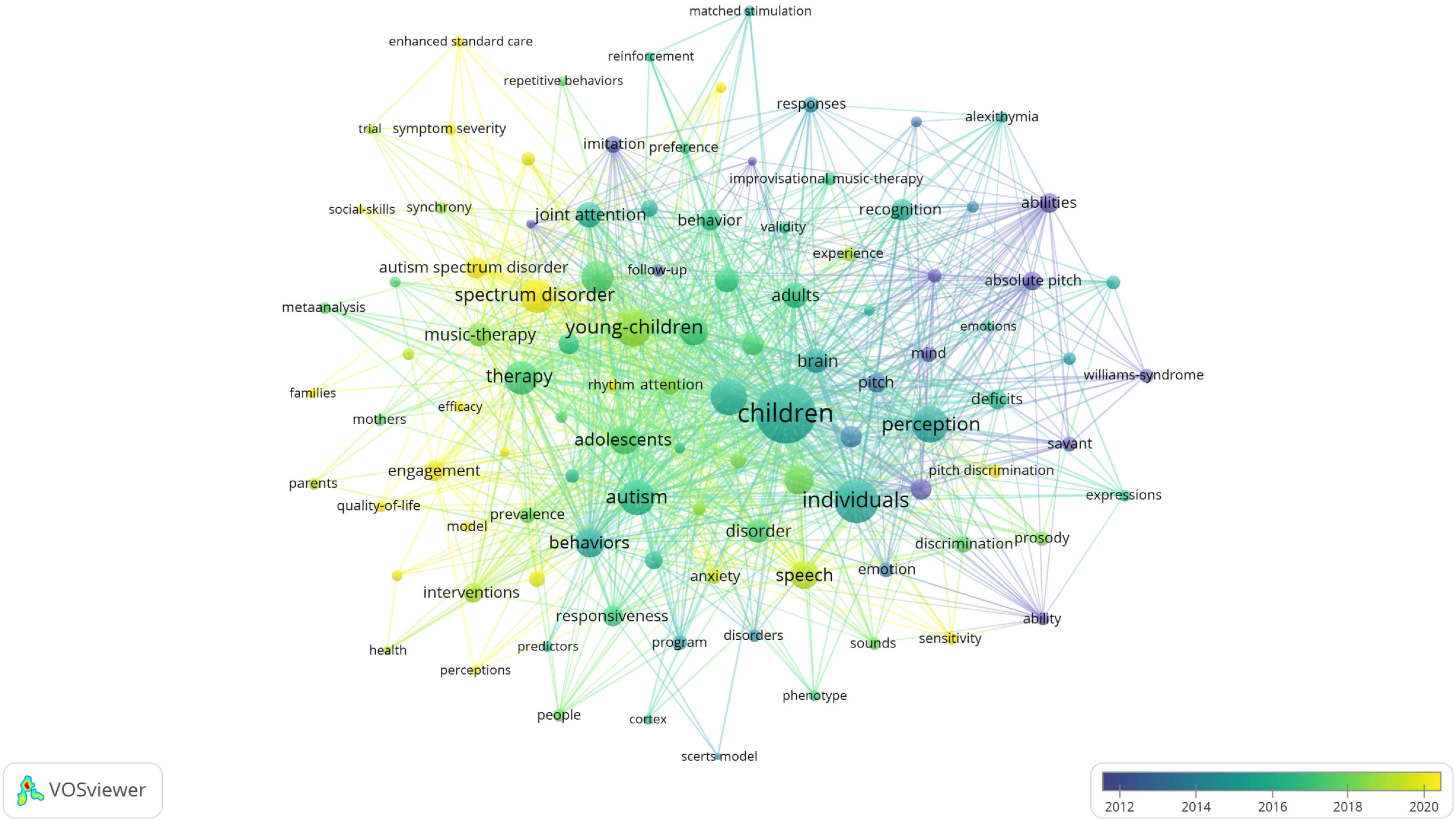
Visual analysis of keyword co-occurrence network analysis.

The most prominent core keywords in the diagram are “children,” “individuals,” “autism,” and “spectrum disorder.” These keywords have the largest nodes, indicating their high frequency in the literature and strong connections with other terms. Keywords such as “therapy,” “engagement,” and “behaviors” are closely linked to “spectrum disorder” and “children,” suggesting these topics are frequently explored together in research. Notably, therapy-related keywords, such as “music therapy” and “joint attention,” form significant networks, indicating the importance of therapeutic interventions in autism research. Based on the color gradient, keywords closer to yellow, such as “engagement,” “interventions,” and “quality of life,” have gained increasing attention in recent years, while those closer to blue, such as “perception” and “absolute pitch,” were more prominent in earlier studies. Children has the highest total link strength (531), followed by individuals (296) and young children (223) (**Figure 7**).

### Analysis of burst keywords

**Figure 8:**
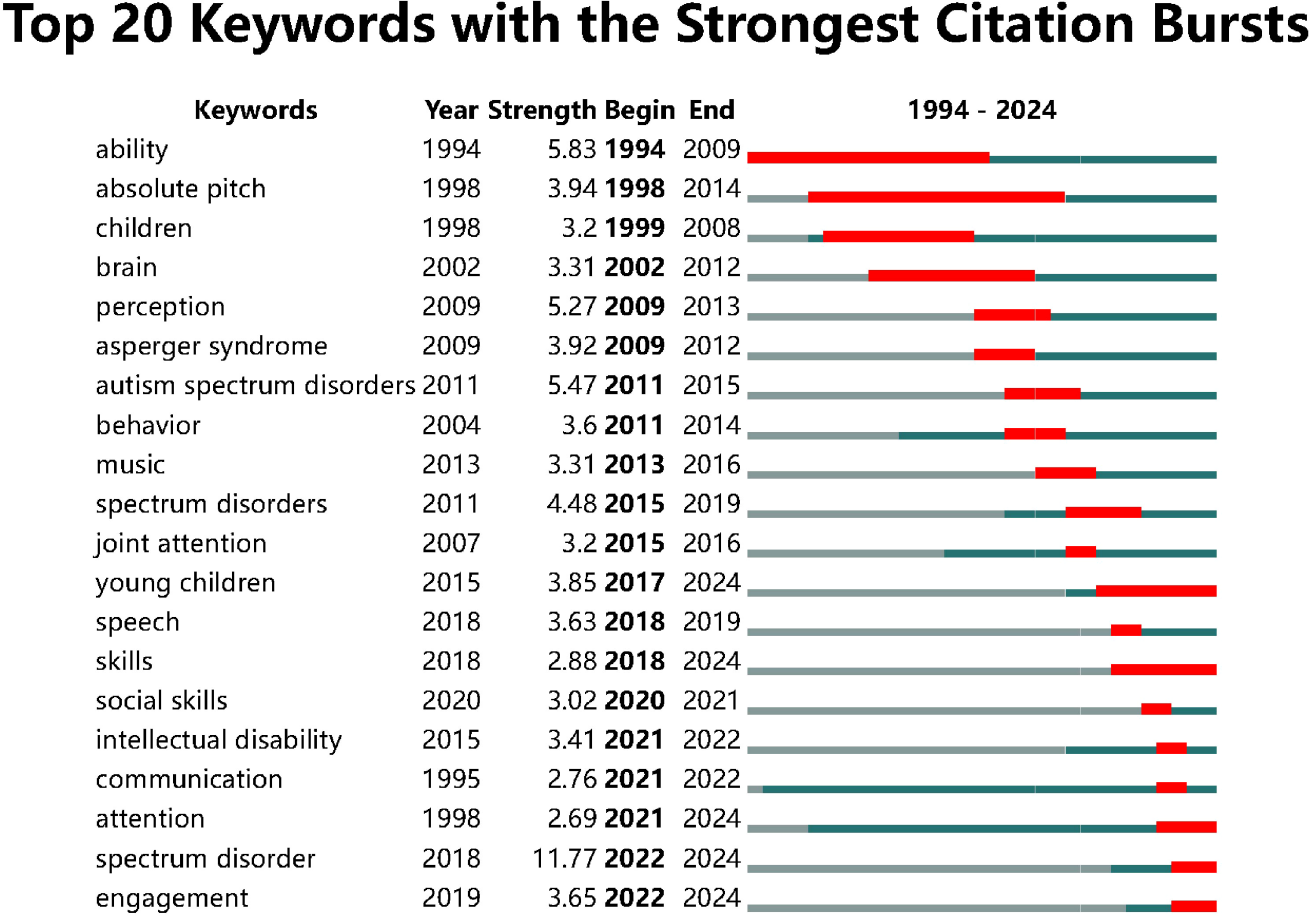
Top 20 keywords with the strongest citation bursts.

The keyword “ability” exhibited the most significant citation burst from 1994 to 2009, with a burst strength of 5.83, indicating a prolonged period of heightened academic focus on this topic. Other keywords with notable burst strengths include “autism spectrum disorders” (burst strength 5.47) from 2011 to 2015 and “spectrum disorder” (burst strength 11.77) from 2022 to 2024, reflecting strong interest in autism-related topics during those years. The keyword “brain” experienced a prolonged citation burst from 2002 to 2012, while “perception” saw a similar surge from 2009 to 2013, suggesting these were areas of sustained research interest. More recently, keywords such as “engagement” (2019-2024), “speech” (2018-2019), and “social skills” (2020-2021) have shown strong citation bursts, indicating that these themes have become significant research hotspots in recent years, particularly in the fields of communication and developmental interventions (**Figure 8**).

## Discussion

Bibliometric methods were used to analyze the growth pattern of research on the influence of music on children with autism spectrum disorder from 2000 to 2024. The analysis indicates two stages of growth based on whether the number of publications exceeds 10 per year for two consecutive years. Before 2010, the research experienced a slow growth phase, with the annual number of publications generally below 10. From 2011 onwards, the field entered a rapid growth stage, with publication numbers consistently exceeding 10 per year. By 2022, the annual publication volume reached a peak of 43, suggesting that research on music’s influence on children with autism spectrum disorder has gained substantial momentum. This increase may be attributed to growing recognition of the therapeutic potential of music interventions for improving social, communication and behavioral skills in children with autism (21, 22). As awareness and interest in these interventions grow, research institutions have likely increased support and funding, further driving the development of this field.

In this field, the *Journal of Music Therapy* has the highest number of publications, totaling 41, indicating its prominence and focus on music-related therapeutic studies. The *Journal of Autism and Developmental Disorders*, with an Impact Factor (IF) of 3.2, has the highest citation count (1,706), reflecting its significant influence and the high impact of its articles on autism. The journal *Autism Research* has the highest Impact Factor (IF 5.3) among the listed journals, highlighting its importance in publishing high-quality autism-related research. JCR (Journal Citation Reports) divides journals into four quartiles (Q1-Q4) based on their Impact Factor. Among the top journals by publication count in this field, Q1 and Q2 journals account for more than 50%. Despite the global nature of research on autism and music therapy, there is limited representation from Asian publishers among these top journals. This underrepresentation indicates a need for developing more internationally influential journals within Asia to better capture the region’s contributions to this field. Establishing such platforms could facilitate a more diverse and comprehensive understanding of music’s impact on autism spectrum disorder.

A total of 153 articles were published by the top-ranking country, the USA, which accounts for 37.23% of all articles. The USA is followed by the UK and Canada, with 51 and 40 articles, respectively. The USA also leads in international collaboration, participating in 77 collaborative projects, followed by the UK with 57 and Norway with 44, indicating a significant emphasis on global partnerships. The dominance of the USA in both publication volume and international collaborations underscores its critical contributions and leadership position in this field, likely influenced by its strong economic conditions and substantial investment in medical research.

The top institutions contributing to research on music interventions for children with autism spectrum disorder are predominantly located in North America and Europe, similar to the distribution of publication numbers by country. Vanderbilt University leads with 41 publications, followed by McGill University with 39 and the University of London with 37. Notably, Canadian institutions such as McGill University and Université de Montréal are highly represented, underscoring Canada’s significant role in this field. In contrast, other countries like China, despite being the fourth in total publication count, do not have as prominent institutional representation in the top tier. The data suggest that institutions with robust international collaboration networks, such as McGill University with 27 collaborations, tend to have higher research output. This highlights the importance of international cooperation for enhancing research competitiveness, particularly for institutions in countries with fewer resources.

Heaton Pamela is the most influential author, with an h-index of 10, having published 14 articles with 138 citations. His articles are primarily published in journals such as the *Annals of the New York Academy of Sciences* (23), *Journal of Autism and Developmental Disorders* (24) and *Research in Developmental Disabilities* (25). He is dedicated to exploring the differences in emotional recognition in music and social contexts among individuals with autism, as well as how music can serve as a unique tool to understand and intervene in the emotional processing difficulties experienced by individuals with autism.

### Research hotspots and frontiers

A keyword co-occurrence network map generated using the VOSviewer tool illustrates the main keywords in autism spectrum disorder research and their temporal evolution, revealing the hotspots and trends in the research field (26). Current research focuses on the effects of music on improving social and communication skills in children with autism. Future studies will explore the long-term impact of music therapy on language, cognition, and behavior in autism children, as well as assess the potential of music to enhance engagement and therapeutic outcomes in educational and treatment settings.

In the earlier time period (2012-2014), research may have focused on the specific phenotypes and musical abilities associated with autism, such as “savant ability” and “absolute pitch.” This suggests that early studies might have explored the correlation between autism and musical capabilities. In 2013, Inge-Marie Eigsti and Deborah A. Fein (27) found that children with optimal outcomes from autism, who no longer show symptoms, do not exhibit the heightened pitch discrimination seen in children with ASD, and this heightened auditory discrimination is linked to both autism symptomatology and language delays. Yasushi Nakai and colleagues (28) conducted a study using a new quantitative acoustic analysis method to assess the speech intonation of children with ASD and children with typical development (TD). The study involved 63 participants (26 with ASD and 37 with TD) who were divided into four groups based on their developmental characteristics and age. The researchers measured the variety in fundamental frequency (F0) patterns using the pitch coefficient of variation (CV), taking into account the mean F0 for each word. The study found that there were no significant differences in pitch CV between ASD and TD groups at preschool age. However, at school age, the TD group exhibited significantly greater pitch CV compared to the ASD group. Additionally, no significant differences were observed between participant types or age groups regarding the range and standard deviation of pitch CV for the entire speech samples. In the ASD group, there was no significant correlation between pitch CV and either the Japanese Autism Screening Questionnaire (ASQ) total score or intelligence quotient levels. However, a significant correlation was found between pitch CV and social reciprocal interaction scores. Future research could further explore the connection between music, emotional expression, social abilities, and cognitive development in autistic individuals, providing a scientific basis for the use of music therapy.

Over time, and particularly between 2014-2016, keywords such as “music-therapy” and “validity” emerged, which may reflect an increased research interest in the efficacy of music therapy in treating certain conditions, such as autism. In 2015, Nandini and colleagues employed a single-subject design to conduct an intervention with three children with autism, aged 3 to 4 years, over 18 sessions, using spoken directives in 9 sessions and sung directives in the other 9. The results indicated that under sung conditions, there was an increase in the frequency of social gestures and eye contact for all participants. The findings suggest that sung directives may serve as a communicative scaffold, helping children with autism to engage more effectively in interactive play activities, enhancing their attention, compliance, and social communication skills (29). Additionally, a study with a randomized controlled trial design was conducted (30), involving a 45-day music therapy intervention for 27 children with mild to moderate autism. The results showed a significant improvement in social skills scores for the experimental group. This study indicates that music therapy, by promoting children’s participation in and enjoyment of music, may help improve their social interaction and communication skills. However, these studies have some limitations. The intervention period in the study was relatively short, and future research could conduct long-term follow-ups to assess the durability of the intervention effects. The specific content and manner of the singing and spoken directives used in the study may affect the results, and future research could further standardize the intervention procedures. The study did not explore in detail the impact of music intervention on other aspects of children with autism (such as language, cognition, or behavior), which are areas that future research could explore.

By the period of 2016-2020, keywords like “autism,” “adolescents,” “interventions,” and “health” became prominent. The focus of research shifts towards the broader application of music and health issues. Authored by Blythe LaGasse et al., the study (31) aimed to assess the impact of music on sensory gating and attention in children with autism. The research involved seven high-functioning autism children, aged 5-12, and seven typically developing children matched for age and gender. It was found that after receiving music therapy, children with autism showed significant improvements in selective attention and a trend towards improvement in sustained attention. Other researchers (32) aim to develop and validate a music-based Autism Diagnostic Scale (MUSAD) for assessing autism in individuals with intellectual disability. However, these studies had a small sample size, and the impact of music on social interaction, language, cognition, or behavior still requires further research.

Based on the results of the burst word analysis, future research may delve deeper into exploring the various aspects of how music affects individuals with spectrum disorders, including its impact on diagnosis, treatment, social adaptability, and associations with other conditions. Individuals with spectrum disorders often face challenges in social skills, so the increase in the keyword “social skills” may indicate that future research will focus more on how music can improve these individuals’ social skills and the role of social skills in the treatment of spectrum disorders. The rise in the keyword “intellectual disability” may suggest that future studies will explore the overlaps and differences between intellectual disabilities and spectrum disorders, as well as how music can better support individuals who have both conditions. As individuals with spectrum disorders face difficulties in communication, the increase in the keyword “communication” may indicate that future research will explore how music can be combined with new communication tools and technologies to help these individuals communicate more effectively. The increase in the keyword “engagement” may suggest that future research will pay more attention to how to improve the participation of music in educational and therapeutic settings, and how to improve the treatment outcomes for individuals with autism by enhancing engagement.

### Strengths and limitations

This bibliometric study is the first to comprehensively explore the distribution trends and major research focuses on the impact of music interventions on children with autism. One of the main strengths of our research is its analysis spanning multiple years, aimed at identifying relevant literature in the field of music interventions for children with autism. However, like previous bibliometric studies, our research also has several limitations. Potential biases include the reliance on citation counts, which may not fully capture an article’s clinical impact. Additionally, the exclusion of non-English publications could limit the scope of the analysis.

## Conclusion

In conclusion, this bibliometric analysis demonstrates that contemporary research predominantly focuses on the effects of music in enhancing social and communication skills among children with autism. Future studies are anticipated to explore the long-term impact of music therapy on language, cognition, and behavior in this population, as well as to assess the potential of music to improve engagement and therapeutic outcomes within educational and treatment contexts.

## Data Availability

The original contributions presented in the study are included in the article/**Supplementary Material**. Further inquiries can be directed to the corresponding author.

## References

[1] Baio J Wiggins L Christensen DL Maenner MJ Daniels J Warren Z . Prevalence of autism spectrum disorder among children aged 8 years - autism and developmental disabilities monitoring Network, 11 Sites, United States, 2014. MMWR Surveill Summ. (2018) 67:1–23. doi: 10.15585/mmwr.ss6706a1, PMID: 29701730 PMC5919599

[2] Muhle R Trentacoste SV Rapin I . The genetics of autism. Pediatrics. (2004) 113:e472–86. doi: 10.1542/peds.113.5.e472, PMID: 15121991

[3] Autism spectrum disorder in under 19s: support and management. In: National Institute for Health and Care Excellence: Clinical Guidelines. London. (2021). 34283415

[4] Dawson G Toth K Abbott R Osterling J Munson J Estes A . Early social attention impairments in autism: social orienting, joint attention, and attention to distress. Dev Psychol. (2004) 40:271–83. doi: 10.1037/0012-1649.40.2.271, PMID: 14979766

[5] Lord C Elsabbagh M Baird G Veenstra-Vanderweele J . Autism spectrum disorder. Lancet. (2018) 392:508–20. doi: 10.1016/S0140-6736(18)31129-2, PMID: 30078460 PMC7398158

[6] Gernsbacher MA Stevenson JL Khandakar S Goldsmith HH . Why does joint attention look atypical in autism? Child Dev Perspect. (2008) 2:38–45. doi: 10.1111/j.1750-8606.2008.00039.x, PMID: 25520747 PMC4266470

[7] Jagadapillai R . Atypical autism: causes, diagnosis and support. Medicina (Kaunas). (2024) 60. doi: 10.3390/medicina60071163, PMID: 39064592 PMC11278543

[8] Kim J Wigram T Gold C . Emotional, motivational and interpersonal responsiveness of children with autism in improvisational music therapy. Autism. (2009) 13:389–409. doi: 10.1177/1362361309105660, PMID: 19535468

[9] Marsh KL Richardson MJ Schmidt RC . Social connection through joint action and interpersonal coordination. Top Cognit Sci. (2009) 1:320–39. doi: 10.1111/j.1756-8765.2009.01022.x, PMID: 25164936

[10] Lim HA . Effect of “developmental speech and language training through music” on speech production in children with autism spectrum disorders. J Music Ther. (2010) 47:2–26. doi: 10.1093/jmt/47.1.2, PMID: 20635521

[11] Rickson DJ Watkins WG . Music therapy to promote prosocial behaviors in aggressive adolescent boys–a pilot study. J Music Ther. (2003) 40:283–301. doi: 10.1093/jmt/40.4.283, PMID: 15015908

[12] Chapin H Jantzen K Kelso JA Steinberg F Large E . Dynamic emotional and neural responses to music depend on performance expression and listener experience. PloS One. (2010) 5:e13812. doi: 10.1371/journal.pone.0013812, PMID: 21179549 PMC3002933

[13] Wan CY Demaine K Zipse L Norton A Schlaug G . From music making to speaking: engaging the mirror neuron system in autism. Brain Res Bull. (2010) 82:161–8. doi: 10.1016/j.brainresbull.2010.04.010, PMID: 20433906 PMC2996136

[14] Garfield E . The history and meaning of the journal impact factor. JAMA. (2006) 295:90–3. doi: 10.1001/jama.295.1.90, PMID: 16391221

[15] Ke L Lu C Shen R Lu T Ma B Hua Y . Knowledge mapping of drug-induced liver injury: A scientometric investigation (2010-2019). Front Pharmacol. (2020) 11:842. doi: 10.3389/fphar.2020.00842, PMID: 32581801 PMC7291871

[16] Lyu K Li J Chen M Li W Zhang W Hu M . A bibliometric analysis of autism spectrum disorder signaling pathways research in the past decade. Front Psychiatry. (2024) 15:1304916. doi: 10.3389/fpsyt.2024.1304916, PMID: 38410675 PMC10895046

[17] Gong XR You XR Guo MR Ding XY Ma BX . Children autism spectrum disorder and gut microbiota: A bibliometric and visual analysis from 2000 to 2023. Med (Baltimore). (2023) 102:e36794. doi: 10.1097/MD.0000000000036794, PMID: 38206702 PMC10754604

[18] van Eck NJ Waltman L . Software survey: VOSviewer, a computer program for bibliometric mapping. Scientometrics. (2010) 84:523–38. doi: 10.1007/s11192-009-0146-3, PMID: 20585380 PMC2883932

[19] Hirsch JE . An index to quantify an individual’s scientific research output. Proc Natl Acad Sci U.S.A. (2005) 102:16569–72. doi: 10.1073/pnas.0507655102, PMID: 16275915 PMC1283832

[20] Bertoli-Barsotti L Lando T . A theoretical model of the relationship between the h-index and other simple citation indicators. Scientometrics. (2017) 111:1415–48. doi: 10.1007/s11192-017-2351-9, PMID: 28596626 PMC5438441

[21] Xia T Li Z . Behavioral training of high-functioning autistic children by music education of occupational therapy. Occup Ther Int. (2022) 2022:6040457. doi: 10.1155/2022/6040457, PMID: 36262376 PMC9553695

[22] Feng H Mahoor MH Dino F . A music-therapy robotic platform for children with autism: A pilot study. Front Robot AI. (2022) 9:855819. doi: 10.3389/frobt.2022.855819, PMID: 35677082 PMC9169087

[23] Molnar-Szakacs I Heaton P . Music: a unique window into the world of autism. Ann N Y Acad Sci. (2012) 1252:318–24. doi: 10.1111/j.1749-6632.2012.06465.x, PMID: 22524374

[24] Heaton P . Age related differences in response to music-evoked emotion among children and adolescents with autism spectrum disorders. J Autism Dev Disord. (2016) 46:1490–1. doi: 10.1007/s10803-015-2671-7, PMID: 26659553

[25] Redondo Pedregal C Heaton P . Autism, music and Alexithymia: A musical intervention to enhance emotion recognition in adolescents with ASD. Res Dev Disabil. (2021) 116:104040. doi: 10.1016/j.ridd.2021.104040, PMID: 34329821

[26] Wu J Chen Y Chen L Ji Z Tian H Zheng D . Global research trends on anti-PD-1/anti-PD-L1 immunotherapy for triple-negative breast cancer: A scientometric analysis. Front Oncol. (2022) 12:1002667. doi: 10.3389/fonc.2022.1002667, PMID: 36713507 PMC9875294

[27] Eigsti IM Fein DA . More is less: pitch discrimination and language delays in children with optimal outcomes from autism. Autism Res. (2013) 6:605–13. doi: 10.1002/aur.1324, PMID: 23929787 PMC3869875

[28] Nakai Y Takashima R Takiguchi T Takada S . Speech intonation in children with autism spectrum disorder. Brain Dev. (2014) 36:516–22. doi: 10.1016/j.braindev.2013.07.006, PMID: 23973369

[29] Paul A Sharda M Menon S Arora I Kansal N Arora K . The effect of sung speech on socio-communicative responsiveness in children with autism spectrum disorders. Front Hum Neurosci. (2015) 9:555. doi: 10.3389/fnhum.2015.00555, PMID: 26578923 PMC4624858

[30] Ghasemtabar SN Hosseini M Fayyaz I Arab S Naghashian H Poudineh Z . Music therapy: An effective approach in improving social skills of children with autism. Adv BioMed Res. (2015) 4:157. doi: 10.4103/2277-9175.161584, PMID: 26380242 PMC4550953

[31] LaGasse AB Manning RCB Crasta JE Gavin WJ Davies PL . Assessing the impact of music therapy on sensory gating and attention in children with autism: A pilot and feasibility study. J Music Ther. (2019) 56:287–314. doi: 10.1093/jmt/thz008, PMID: 31225588

[32] Bergmann T Heinrich M Ziegler M Dziobek I Diefenbacher A Sappok T . Developing a diagnostic algorithm for the music-based scale for autism diagnostics (MUSAD) assessing adults with intellectual disability. J Autism Dev Disord. (2019) 49:3732–52. doi: 10.1007/s10803-019-04069-y, PMID: 31161304

