# Supplementary material for: Research trends of music in children with autism: a bibliometric analysis
Source: Front Psychiatry. 2025 Aug 12;16:1553883. doi: 10.3389/fpsyt.2025.1553883 (PMC12379730; doi:10.3389/fpsyt.2025.1553883)
Supplement: Supplementary file 1 [file Table1.docx]

**Table 1 Publication and Citation Profiles of Leading Countries**

| **Country** | **Articles** | **Freq** | **MCP_Ratio** | **TP** | **TP_rank** | **TC** | **TC_rank** | **Average Citations** |
| --- | --- | --- | --- | --- | --- | --- | --- | --- |
| USA | 153 | 37.23 | 13.73 | 381 | 1 | 4187 | 1 | 27.4 |
| UK | 51 | 12.41 | 23.53 | 130 | 2 | 1356 | 3 | 26.6 |
| CANADA | 40 | 9.73 | 42.50 | 123 | 3 | 1581 | 2 | 39.5 |
| CHINA | 25 | 6.08 | 32.00 | 68 | 4 | 152 | 9 | 6.1 |
| AUSTRALIA | 17 | 4.14 | 23.53 | 39 | 5 | 448 | 4 | 26.4 |
| ISRAEL | 12 | 2.92 | 25.00 | 33 | 8 | 81 | 13 | 6.8 |
| NORWAY | 12 | 2.92 | 58.33 | 35 | 6 | 290 | 6 | 24.2 |
| GERMANY | 11 | 2.68 | 45.45 | 34 | 7 | 204 | 8 | 18.5 |
| DENMARK | 6 | 1.46 | 66.67 | 18 | 11 | 285 | 7 | 47.5 |
| ITALY | 6 | 1.46 | 33.33 | 30 | 9 | 99 | 10 | 16.5 |
| JAPAN | 6 | 1.46 | 16.67 | 16 | 12 | 63 | 16 | 10.5 |
| KOREA | 6 | 1.46 | 33.33 | 16 | 13 | 341 | 5 | 56.8 |
| IRAN | 5 | 1.22 | 60.00 | 14 | 14 | 87 | 11 | 17.4 |
| TURKEY | 5 | 1.22 | 0.00 | 12 | 15 | 16 | 27 | 3.2 |
| FRANCE | 4 | 0.97 | 100.00 | 19 | 10 | 72 | 15 | 18.0 |
| IRELAND | 4 | 0.97 | 25.00 | 11 | 16 | 55 | 17 | 13.8 |
| NETHERLANDS | 4 | 0.97 | 0.00 | 10 | 17 | 20 | 24 | 5.0 |
| SPAIN | 4 | 0.97 | 0.00 | 9 | 21 | 1 | 37 | 0.2 |
| BRAZIL | 3 | 0.73 | 33.33 | 9 | 19 | 84 | 12 | 28.0 |
| INDIA | 3 | 0.73 | 66.67 | 9 | 20 | 75 | 14 | 25.0 |

Note(s): Articles: Publications of Corresponding Authors only. Freq: Frequence of Total Publications. MCP_Ratio: Proportion of Multiple Country Publications. TP: Total Publications. TP_rank: Rank of Total Publications. TC: Total Citations. TC_rank: Rank of Total Citations. Average Citations: The average number of citations per publication.

**Table 2 Bibliometric Indicators of High-Impact Journals**

| Journal | H_index | IF | JCR_Quartile | PY_start | TP | TP_rank | TC | TC_rank |
| --- | --- | --- | --- | --- | --- | --- | --- | --- |
| JOURNAL OF AUTISM AND DEVELOPMENTAL DISORDERS | 19 | 3.2 | Q1 | 1979 | 36 | 2 | 1706 | 1 |
| JOURNAL OF MUSIC THERAPY | 19 | 1.9 | Q3 | 1984 | 41 | 1 | 661 | 2 |
| AUTISM | 11 | 5.2 | Q1 | 2007 | 14 | 5 | 421 | 4 |
| NORDIC JOURNAL OF MUSIC THERAPY | 9 | 1.6 | Q2 | 2011 | 26 | 3 | 147 | 17 |
| RESEARCH IN AUTISM SPECTRUM DISORDERS | 9 | 2.2 | Q1 | 2008 | 12 | 8 | 236 | 6 |
| AUTISM RESEARCH | 7 | 5.3 | Q1 | 2010 | 14 | 6 | 169 | 14 |
| JOURNAL OF APPLIED BEHAVIOR ANALYSIS | 7 | 2.9 | Q1 | 2009 | 9 | 10 | 344 | 5 |
| FRONTIERS IN PSYCHOLOGY | 6 | 2.6 | Q2 | 2015 | 17 | 4 | 204 | 8 |
| ARTS IN PSYCHOTHERAPY | 5 | 1.5 | Q3 | 2012 | 13 | 7 | 91 | 29 |
| BEHAVIOR MODIFICATION | 5 | 2 | Q3 | 2009 | 5 | 14 | 59 | 55 |
| MUSIC PERCEPTION | 5 | 1.3 | Q4 | 1998 | 7 | 11 | 195 | 10 |
| BEHAVIORAL INTERVENTIONS | 4 | 1.1 | Q3 | 2010 | 6 | 13 | 69 | 46 |
| CHILD NEUROPSYCHOLOGY | 4 | 1.6 | Q3 | 2004 | 5 | 15 | 63 | 50 |
| PLOS ONE | 4 | 2.9 | Q1 | 2010 | 7 | 12 | 221 | 7 |
| PSYCHOLOGY OF MUSIC | 4 | 1.6 | Q3 | 2012 | 10 | 9 | 117 | 20 |
| ANNALS OF THE NEW YORK ACADEMY OF SCIENCES | 3 | 4.1 | Q1 | 2018 | 4 | 17 | 192 | 11 |
| FRONTIERS IN NEUROSCIENCE | 3 | 3.2 | Q2 | 2014 | 4 | 18 | 57 | 56 |
| INTERNATIONAL JOURNAL OF DEVELOPMENTAL DISABILITIES | 3 | 1.5 | Q3 | 2019 | 4 | 19 | 11 | 259 |
| OCCUPATIONAL THERAPY INTERNATIONAL | 3 | 1.3 | Q3 | 2009 | 4 | 20 | 7 | 411 |
| PHILOSOPHICAL TRANSACTIONS OF THE ROYAL SOCIETY B-BIOLOGICAL SCIENCES | 3 | 5.4 | Q1 | 2009 | 3 | 24 | 101 | 25 |

Note(s): H_index: The H-index of the journal, which measures both the productivity and citation impact of the publications. IF: Impact Factor, indicating the average number of citations to recent articles published in the journal. JCR_Quartile: The quartile ranking of the journal in the Journal Citation Reports, indicating the journal's ranking relative to others in the same field (Q1: top 25%, Q2: 25%-50%, Q3: 50%-75%, Q4: bottom 25%). PY_start: Publication Year Start, indicating the year the journal started publication. TP: Total Publications. TP_rank: Rank of Total Publications. TC: Total Citations. TC_rank: Rank of Total Citations.

**Table 3 Publication and Citation Profiles of High-Impact Authors**

| **Authors** | **H_index** | **G-index** | **M-index** | **PY_start** | **TP** | **TP_Frac** | **TP_rank** | **TC** | **TC_rank** |
| --- | --- | --- | --- | --- | --- | --- | --- | --- | --- |
| HEATON PAMELA | 10 | 14 | 0.556 | 2007 | 14 | 5.55 | 1 | 138 | 4 |
| GOLD CHRISTIAN | 9 | 12 | 0.529 | 2008 | 12 | 2.50 | 2 | 225 | 1 |
| ALLEN RORY | 6 | 6 | 0.353 | 2008 | 6 | 2.07 | 11 | 75 | 11 |
| ELEFANT COCHAVIT | 6 | 8 | 0.600 | 2015 | 8 | 2.38 | 6 | 75 | 12 |
| GERETSEGGER MONIKA | 6 | 7 | 0.462 | 2012 | 7 | 1.25 | 8 | 94 | 8 |
| HEATON P | 6 | 6 | 0.222 | 1998 | 6 | 3.33 | 12 | 172 | 2 |
| LANOVAZ MARC J. | 6 | 8 | 0.375 | 2009 | 8 | 2.81 | 7 | 34 | 49 |
| RAPP JOHN T. | 6 | 6 | 0.375 | 2009 | 6 | 2.14 | 14 | 29 | 59 |
| SHARDA MEGHA | 6 | 9 | 0.600 | 2015 | 9 | 1.56 | 4 | 73 | 13 |
| BELLUGI URSULA | 5 | 5 | 0.333 | 2010 | 5 | 0.78 | 16 | 15 | 121 |
| LENSE MIRIAM D. | 5 | 6 | 0.357 | 2011 | 6 | 1.54 | 13 | 19 | 96 |
| LEVITIN DANIEL J. | 5 | 5 | 0.313 | 2009 | 5 | 1.02 | 20 | 40 | 28 |
| SILVERMAN MICHAEL J. | 5 | 7 | 0.385 | 2012 | 9 | 4.17 | 5 | 19 | 97 |
| BERGMANN THOMAS | 4 | 5 | 0.400 | 2015 | 5 | 1.01 | 17 | 10 | 158 |
| FOMBONNE ERIC | 4 | 4 | 0.250 | 2009 | 4 | 0.77 | 23 | 40 | 26 |
| HERMELIN B | 4 | 4 | 0.148 | 1998 | 4 | 1.33 | 24 | 107 | 6 |
| HOLCK ULLA | 4 | 5 | 0.308 | 2012 | 5 | 1.31 | 18 | 48 | 18 |
| JIANG CUNMEI | 4 | 7 | 0.400 | 2015 | 7 | 1.35 | 9 | 22 | 81 |
| KIM JINAH | 4 | 5 | 0.235 | 2008 | 5 | 1.88 | 19 | 168 | 3 |
| LIU FANG | 4 | 8 | 0.400 | 2015 | 9 | 1.79 | 3 | 23 | 75 |

Note(s): H_index: The H-index of the journal, which measures both the productivity and citation impact of the publications. G_index: The G-index of the journal, which gives more weight to highly-cited articles. M_index: The m-index of the journal, which is the H-index divided by the number of years since the first published paper. PY_start: Publication Year Start, indicating the year the journal started publication. TP: Total Publications. TP_Frac: Total Publications Fractionalized. TP_rank: Rank of Total Publications. TC: Total Citations. TC_rank: Rank of Total Citations.
